# Hunting mode and habitat selection mediate the success of human hunters

**DOI:** 10.1186/s40462-024-00471-z

**Published:** 2024-04-16

**Authors:** Kaitlyn M. Gaynor, Alex McInturff, Briana L. Abrahms, Alison M. Smith, Justin S. Brashares

**Affiliations:** 1 Departments of Zoology and Botany, University of British Columbia, Vancouver, BC Canada; 2 U.S. Geological Survey Washington Cooperative Fish and Wildlife Research Unit, School of Environmental and Forest Sciences, University of Washington, Seattle, WA USA; 3 Center for Ecosystem Sentinels, Department of Biology, University of Washington, Seattle, WA USA; 4 Hopland Research and Extension Center, University of California, Division of Agriculture and Natural Resources, Hopland, CA USA; 5 Department of Environmental Science, Policy, and Management, University of California - Berkeley, Berkeley, CA USA

**Keywords:** Movement ecology, Predator–prey interactions, Hunting mode, Habitat domain, Human ecology, Human dimensions

## Abstract

**Background:**

As a globally widespread apex predator, humans have unprecedented lethal and non-lethal effects on prey populations and ecosystems. Yet compared to non-human predators, little is known about the movement ecology of human hunters, including how hunting behavior interacts with the environment.

**Methods:**

We characterized the hunting modes, habitat selection, and harvest success of 483 rifle hunters in California using high-resolution GPS data. We used Hidden Markov Models to characterize fine-scale movement behavior, and k-means clustering to group hunters by hunting mode, on the basis of their time spent in each behavioral state. Finally, we used Resource Selection Functions to quantify patterns of habitat selection for successful and unsuccessful hunters of each hunting mode.

**Results:**

Hunters exhibited three distinct and successful hunting modes (“coursing”, “stalking”, and “sit-and-wait”), with coursings as the most successful strategy. Across hunting modes, there was variation in patterns of selection for roads, topography, and habitat cover, with differences in habitat use of successful and unsuccessful hunters across modes.

**Conclusions:**

Our study indicates that hunters can successfully employ a diversity of harvest strategies, and that hunting success is mediated by the interacting effects of hunting mode and landscape features. Such results highlight the breadth of human hunting modes, even within a single hunting technique, and lend insight into the varied ways that humans exert predation pressure on wildlife.

**Supplementary Information:**

The online version contains supplementary material available at 10.1186/s40462-024-00471-z.

## Background

From the Pleistocene to the present, the hunting of wild animals by humans has altered ecosystems [1, 2]. The human species occupies a novel trophic position as a globally widespread apex predator, with unprecedented lethal and non-lethal effects on prey and ecosystems [3, 4]. Simultaneously, hunting also provides benefits to human societies and an essential tool for the management of wild animal populations, especially in the absence of large carnivores [5–7]. While hunting is widely recognized for its relevance to management and conservation, we have a more limited understanding of the ecological mechanics of contemporary hunting. The science and management of hunting has historically focused on its broad-scale numerical effects on prey species (e.g., [8, 9]). There has been far less research on the fine-scale movement and behavior of hunters and the effects of such behavioral choices on hunting success [8, 10, 11].

With recent advances in GPS technology and analytical methods from movement ecology, we are increasingly able to characterize the fine-scale spatial behavior and hunting modes of non-human predators [12–15]. Across non-human predator species, distinct hunting modes interact with features of the physical landscape to shape lethal and non-lethal effects of predation [16]. A predator’s hunting mode is the typical behavioral strategy that it uses to find and capture prey [17]. Some predators employ “coursing” or “active” hunting modes, in which they move throughout the landscape in search of prey, and initiate an attack upon encounter [18]. Other predators employ “ambush” or “sit-and-wait” strategies, generally remaining stationary and relying on surprise attacks of prey that come upon them [19, 20]. Finally, some predators exhibit “stalking” or “sit-and-pursue” hunting modes, in which predators pursue prey over shorter distances [21]. Although individual animals may exhibit a range of behaviors in the context of hunting (i.e., moving, sprinting/jumping, hiding), the characterization of dominant hunting modes has revealed important differences in the ecological effects of predators [22].

A predator’s hunting mode can influence its “habitat domain,” defined as the spatial area that a predator uses when hunting [23]. The environmental characteristics of a predator’s habitat domain may vary based on the predator’s hunting mode, as a given hunting mode tends to be most effective in certain habitats [24]. For example, sit-and-wait or stalking predators may rely more on dense vegetative cover or other landscape features in which to hide. Meanwhile, given that coursing predators rely on chase, they may hunt in flat, open areas [18]. The relative availability of suitable hunting habitat will determine the breadth of a predator’s habitat domain, with implications for prey risk trade-offs and predation rates and patterns. The combination of fine-scale predator movement data, environmental features, and hunting success can therefore shed light on the mechanistic links between predator behavior and predation patterns across landscapes.

Humans are unique predators, given that we exhibit a diversity of hunting strategies as a species [25]. The wide range of human hunting technologies, target prey species, and accessible habitats has generated a wide variety of approaches to hunting. Even within a single technology (e.g., rifles) at a given site, hunters frequently adopt different behavioral strategies for hunting [25]. While many aspects of human hunting have no parallels in wild predators, there is evidence that distinct behavioral strategies of hunters have different ecological effects, analogous to wild predators with different hunting modes [26–28]. Studying human predators therefore offers a unique opportunity to understand variation in hunting modes within a species, and how these hunting modes interact with the landscape to influence patterns of harvest success [26] and non-consumptive effects [27, 28]. Methodologically, it is also easier to study entire populations of human hunters and gather fine-scale data on their movements and hunting success, offering insights that are not typically feasible for other predator species. By studying a behaviorally complex predator at high resolutions and sample sizes, research on the behavioral ecology of humans can offer insights that simultaneously advance our understanding of predator–prey ecology and of human-wildlife interactions.

Previous studies have demonstrated that hunter use of the landscape is patterned in space, and that landscape features thus lead to variation in hunting pressure experienced by prey [26, 29] and in hunter success [30–33]. Our research builds on prior studies by examining the behavioral mechanisms underlying these spatial patterns, including the factors that motivate changes in hunter behavior, the hunting modes that emerge from behavioral patterns, and the connections between hunter behavior and success. Here, we tracked the movements of rifle hunters in pursuit of black-tailed deer (*Odocoileus hemionus columbianus*) in California. We recognize that human hunting varies widely in its goals, magnitude, and impacts, depending on the sociocultural and ecological contexts, and while our approach has broader utility to the study of hunter behavior, we limit our inferences here to this study system. Deer hunting in California today is primarily a recreational rather than a subsistence activity, and it is predominantly characterized by the use of modern firearms. We took advantage of a public deer hunt on a research station in California to track every hunter on the site, representing in high-resolution, near-complete coverage of all hunters in the study area over seven hunting seasons.

Our objectives were to (1) characterize the hunting modes of all rifle hunters, based on characteristics of movement trajectories; (2) compare spatiotemporal patterns of harvest and habitat selection across hunting modes; and (3) understand how hunting mode and landscape features interact to influence harvest success. We predicted that hunters that exhibited ambush or sit-and-wait strategies would select for more closed habitats, while hunters using coursing or stalking strategies would select for more open habitats. We predicted that these selection patterns would be stronger for the more successful hunters, given that efficiency and effectiveness may be stronger if hunters use a narrower habitat domain that best facilitates their particular hunting mode.

## Methods

To understand patterns of hunter movement behavior, we collected GPS tracks of all hunters in the study area (2015–2022). We first classified the behavioral state of each location for each hunter, using Hidden Markov Models (HMMs; location-level classification of behavior). Next, we used k-means clustering to group hunters into distinct hunting modes based on the relative time that they spent in each behavioral state (hunter-level classification of behavioral strategy). Finally, we used Resource Selection Functions (RSFs) to evaluate patterns of habitat selection for each hunting mode, comparing habitat selection between successful and unsuccessful hunters.

### Study area

**Fig. 1:**
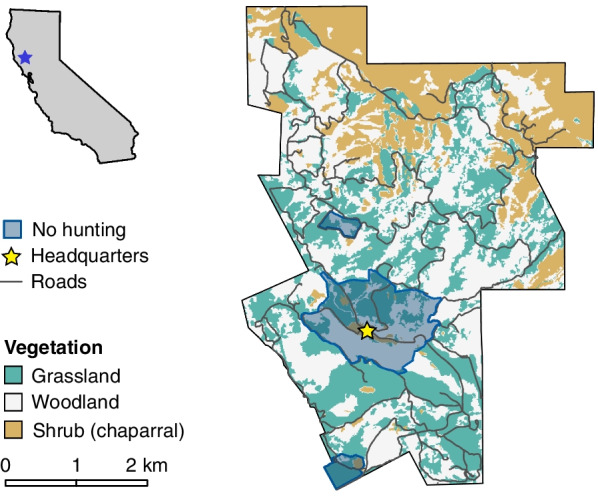
The Hopland Research and Extension Center study area in California, USA where we tracked 483 rifle hunters (2015–2022). Two small pastures and the area surrounding site headquarters are off limits to hunters

We conducted primary data collection at the 2,168-hectare Hopland Research and Extension Center (HREC) in Mendocino County, California (Latitude: 39.002, Longitude: -123.084; Fig. 1). The site features habitat types including grassland, oak woodland, and chaparral, with a network of dirt roads and fences. Elevation at the site ranges from 162 to 929 m above sea level, and includes flat pastures, rolling hills, and steep mountains and canyons. Genetic capture-recapture monitoring of deer on the site reveals a high density of deer (38 deer/km^2^) throughout the study area; deer are very abundant in the study area relative to other nearby sites [34]. The site hosts an annual public hunt, in which twenty hunters per day are selected by lottery from a pool of applicants, for 4–6 days each year. In 2020, a restricted multi-day hunt was introduced for a small number of hunters. More details on the hunt are provided in Additional file 1: Table S1.

### Data collection

Our study took place each August–September from 2015 to 2022, excluding 2018 due to wildfire. We invited all hunters at the study site to participate in our study (Additional file 1: Table S1). We had a 100% rate of participation (n = 483 hunters representing 648 hunter-days).

We provided each hunter with a GPS unit (i-gotU GT-600) that was programmed to take a GPS fix every 5 s from 5am to 10 pm to encompass legal hunting hours at the study site. We asked hunters to keep the GPS unit in a pocket that would remain on their person, even when they were moving on foot. All harvested deer were brought back to headquarters, and we confirmed with the hunters whether each logger was associated with a successful or unsuccessful hunt. Upon data retrieval, we resampled all tracks to a fix rate of 3 min to accommodate GPS error and computational limitations. We followed data cleaning procedures described in detail in the supplementary methods.

### Spatial data

We identified environmental features that we a priori hypothesized to influence hunter behavior and habitat use: distance to nearest road, ruggedness, viewshed, and density of each of the three habitat types (woodland, grassland, and chaparral). These hypotheses were drawn from existing literature on human hunter movement and behavior. We generated raster layers for each feature in the study area. Additional details on the development of spatial variables are provided in the supplementary methods section. We extracted spatial covariates at each point, and we calculated the elapsed time since sunrise for each point using the suncalc package [35]. We standardized all covariates prior to modeling.

### Behavioral state classification with Hidden Markov Models

To identify fine-scale behaviors of hunters, we used the moveHMM package [36] to fit a hidden Markov model to the hunter movement data. We ran a global model with all predictors (distance to road, viewshed, ruggedness, woodland density, chaparral density, and time since sunrise). We assigned movement points to one of three behavioral states, as initial modeling indicated that three-state models performed better than two-state models (based on Akaike Information Criterion (AIC)), and best corresponded to self-described hunter behavior. We interpreted State 1 as corresponding to a stationary state (searching, resting, or processing deer), State 2 to walking on foot, and State 3 to driving in a vehicle.

We followed best practices when choosing initial parameter values [36]. We included a zero-mass parameter for step length given the high proportion of step lengths equal to 0 (17% of all steps). To determine whether our models were sensitive to initial parameter choice, we ran 100 iterations of the model with randomly-chosen starting parameters for step length mean, step length standard deviation, step length zero mass, and turning angle concentration. Our model converged on the same parameters for 82 of 100 of the iterations, and this model had the maximum likelihood, indicating numerical stability. We then used the parameter values from the best model as our starting values for all subsequent modeling. Based on the global model, we determined the most probable behavioral state at each step for each hunter, and determined the percentage of time that each hunter spent in each of the behavioral states. Following identification of the three states from movement parameters, we further distinguished between resting behavior on road (< 10 m from road) and off road (> 10 m from road), as these behaviors are associated with different hunting strategies (resting on the road to visually scan for deer on the landscape vs. resting off the road in a sit-and-wait hunting strategy).

### Identification of distinct hunting modes

To identify the dominant hunting mode of each hunter, we used k-means clustering to group hunters on the basis of their time spent in each fine-scale behavioral state. We determined the optimal value of k using the elbow method heuristic. Specifically, we plotted the total within-cluster sum of squares as a function of k, and determined the value of k at which this sum of squares began declining linearly.

We then ran logistic regressions to evaluate the effect of hunting mode on harvest success. Additional model covariates included year (as we were interested in whether hunting success changed over time) and whether the track came from a single-day or multi-day hunter. We tested all possible covariate combinations and we also explored interactions among hunting mode and the other covariates, to examine whether the effectiveness of different hunting modes changed over time, or varied between single- and multi-day hunts. We compared models using AIC. We also determined relative variable importance (RVI), as calculated by summing the Akaike weights of all models in which the variables appeared.

We also evaluated whether the time of day at which deer were harvested varied across hunting modes, for hunters for which we had known harvest times (n = 37 of 39 successful tracked hunters). We compared harvest time (elapsed time since sunrise) for each of the three clusters using an Anderson–Darling test, a non-parametric rank test of whether samples from different groups came from the same distribution [37].

### Evaluating habitat selection

To evaluate patterns of habitat selection by hunters using different hunting modes, and to evaluate connections between habitat selection and harvest success, we used Resource Selection Functions (RSFs). RSFs compare environmental features of used versus available locations in a logistic regression [38]. We compared locations recorded by hunter GPS trackers (used locations) to locations that we systematically sampled throughout the huntable area at a 30 × 30 m resolution. To evaluate potential links between habitat selection and hunting success, we ran separate models for successful and unsuccessful hunters in each of the hunting modes, and we used the same predictors in all models to facilitate comparison of model coefficients. Model covariates included the same spatial covariates used in the HMM: ruggedness, viewshed, chaparral density, and woodland density. We assigned a weight of 5,000 to the available points, and 1 to the used points, following [39]. We first ran mixed models with a random intercept for track ID, but among-individual variance was 0 for all models, resulting in a singular fit. We therefore removed the random intercept to ensure estimate stability. In addition, because RSFs assume spatiotemporal independence between points, we checked the effect of fix interval. We thinned the “used” points to a 30 min interval to reduce spatial autocorrelation between points, while retaining sufficient data for each individual hunter (mean of 20.3 used points per hunter). Conclusions remained unchanged despite the tenfold reduction in fix rate, and the results of this model are presented in the supplementary material (Additional file 1: Fig. S10, Table S5).

To rule out any potential issues of circularity when using some of the same spatial covariates to classify behavior (which was then used to identify hunting mode) and to compare differences in habitat selection across hunting modes, we also re-ran the HMMs without any spatial covariates, classifying behavior based only on step length and turn angle. We then re-ran the k-means clustering analysis and RSFs with the updated behavior, and conclusions again remained unchanged (Additional file 1: Fig. S11). We have chosen to retain the spatial covariates in the HMM for all analyses presented here, to improve the accuracy of behavioral classification (particularly with regard to driving). The HMM uses information about the spatial covariates to improve predictions of behavioral states at a given location, while the RSF compares all used locations (regardless of behavioral state) to a random set of available points within the study area to evaluate patterns of habitat selection.

## Results

Out of all 648 hunter-days at the Hopland Research and Extension Center from 2015–2022, there were 95 successful harvests of deer (15% success rate). We successfully recovered tracks from 483 hunter-days, and of these, 62 tracks corresponded to hunters that harvested a deer (henceforth, "successful hunters"). Some logger-days were not recorded due to equipment malfunction and were excluded from our analysis.

The Hidden Markov Models revealed that the probability of transitioning among the three behavioral states (stationary, driving, and walking; Additional file 1: Figs. S3, S4) varied as a function of the spatial and temporal covariates included in our model (Additional file 1: Figs. S5, S6). We found that, across all individuals, hunters spent an average of 31% (standard deviation SD: 17%) of time in the stationary state (14% on-road [SD: 10%], and 16% off-road [SD: 14%]), 26% of time moving on foot (SD: 15%), and 44% of time searching by vehicle (SD: 20%). Model pseudoresiduals for step length and turn angle were normally distributed, indicating suitable model fit.

**Fig. 2:**
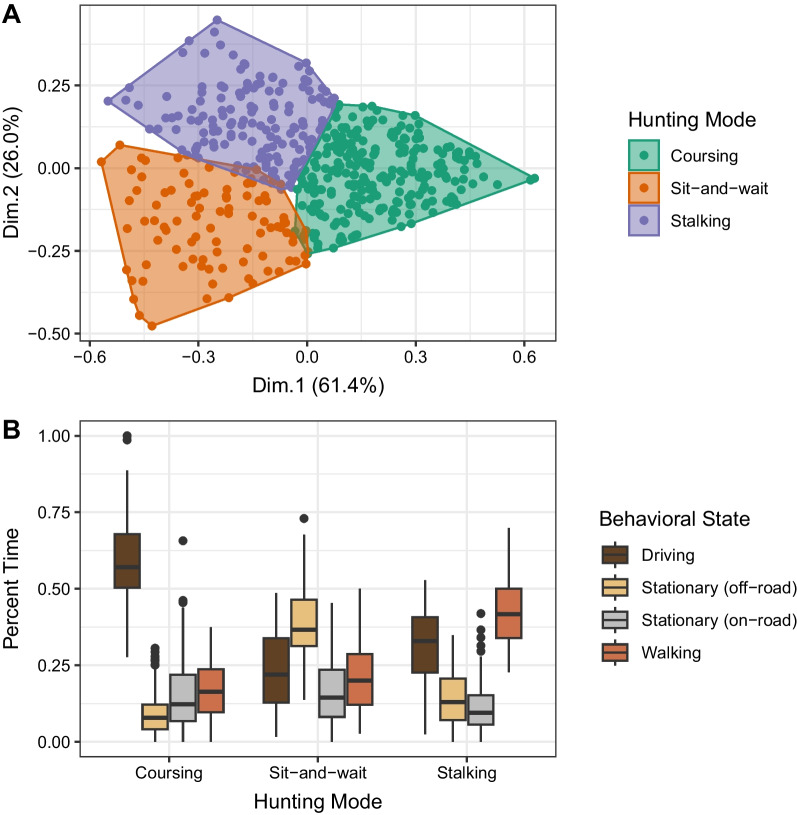
**A** Biplot showing distribution of deer hunters across hunting mode clusters, using the first two dimensions of a Principal Components Analysis based on relative time spent in each behavioral state. PCA was conducted for visualization purposes here. **B** Average time spent in each behavioral state for rifle hunters across hunting modes. Behavioral states were identified using Hidden Markov Models, and hunting modes were identified with k-means clustering. Box plots depict the minimum, first quartile, median, third quartile, and maximum, with outliers depicted as single points

We identified three clusters of hunting mode, by k-means clustering based on the relative time spent in each behavioral state (Fig. 2, Additional file 1: Figs. S7, S8). A “coursing” mode (n = 240 hunters) was characterized by mostly driving behavior with very little time spent stationary off-road; “stalking” (n = 149) consisted of relatively more walking behavior as compared to the other hunting modes; and “sit-and-wait” (n = 94) was characterized by more off-road stationary behavior.

**Fig. 3:**
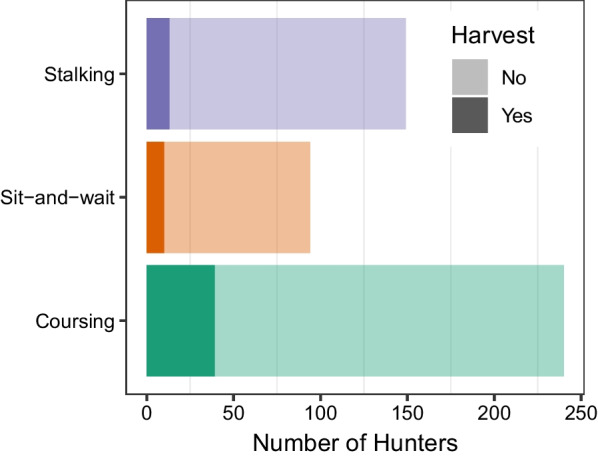
Frequency of successful and unsuccessful rifle hunters by hunting mode for deer. Coursing was the most common and most successful of the three hunting modes (in both absolute and relative terms). Sit-and-wait hunters were the rarest, and stalking hunters were the least successful. Success rates were 9% for stalking hunters, 11% for sit-and-wait hunters, and 16% for coursing hunters

Hunting mode was an important predictor of harvest success (RVI = 0.63; Additional file 1: Table S2, Fig. S9), and success was greater for the coursing hunting mode than for stalking or sit-and-wait. The harvest success rate was 16.3% for "coursing," 8.7% for "stalking," and 10.6% for "sit-and-wait” (Fig. 3). However, the most important predictor of success was whether the track came from a single or multi-day hunt, with odds of success 3.33 times higher for multi-day hunt tracks (95% CI of Odds Ratio 1.42–7.37; RVI = 0.87; Additional file 1: Table S2, Fig. S9). Year was relatively less important (RVI = 0.27), although there was a slight trend towards higher harvest success over time (Additional file 1: Table S2, Fig. S9).

**Fig. 4:**
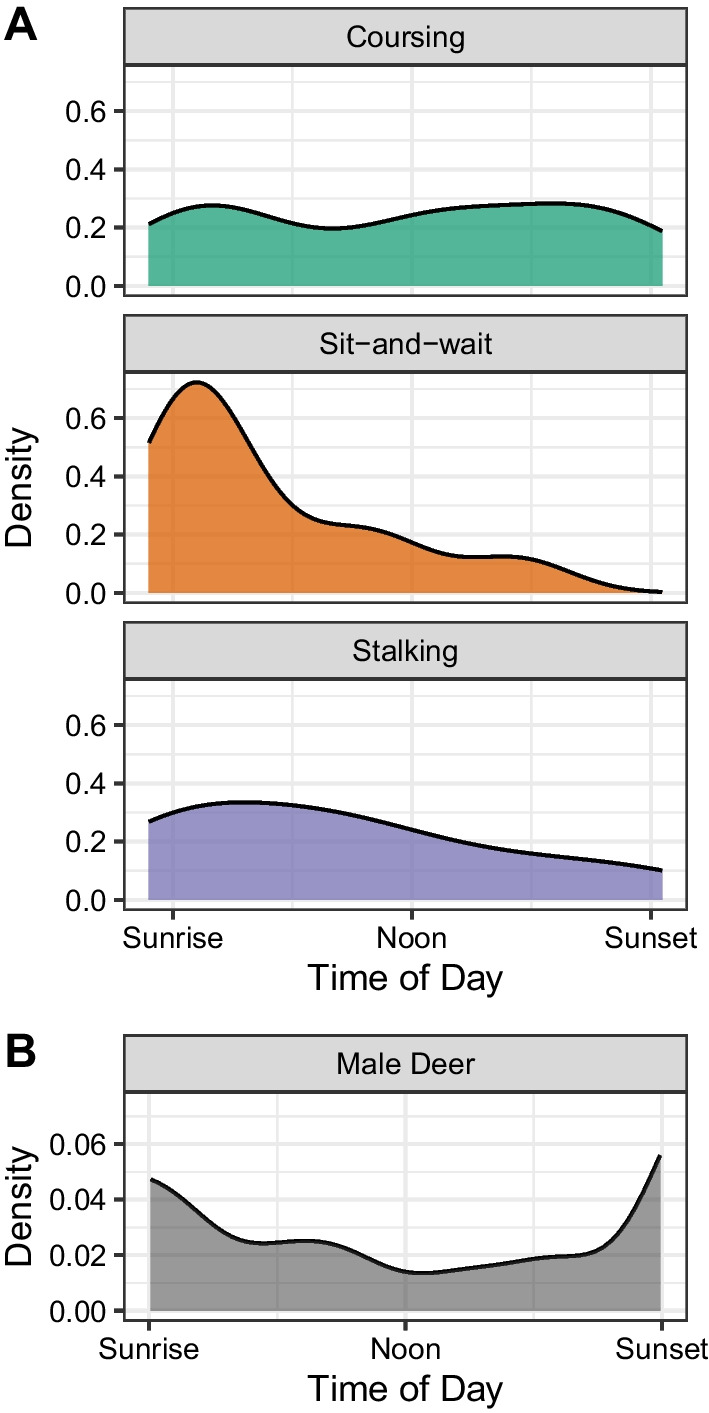
The time of day of deer harvest varied among the three hunting modes used by human hunters. **A** Density of harvest times, scaled by local sunrise and sunset times, for each hunting mode. **B** Density of legal buck camera trap records (n = 138) during four hunting seasons (2016–2020) in the Hopland Research and Extension Center study area in California, USA study area

The time of day at which deer were harvested varied across the three hunting modes (Fig. 4; Anderson–Darling test; AD = 3.98, P = 0.05). Harvest by sit-and-wait hunters clustered in the early morning soon after sunrise when male deer were most active, while coursing hunters had relatively more success in the later part of the day compared to stalking or sit-and-wait hunters.

**Fig. 5:**
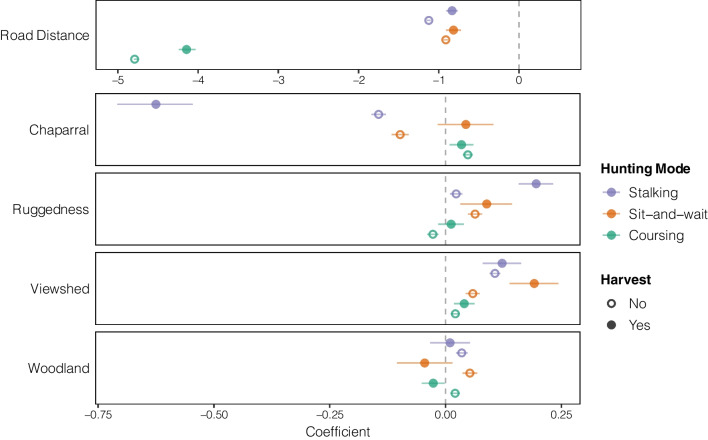
Habitat selection patterns of rifle hunters at the Hopland Research and Extension Center, California. Points represent coefficients from Resource Selection Function (RSF) models (with 95% confidence intervals) for each of the three hunting modes (coursing, sit-and-wait, stalking), run separately for successful and unsuccessful hunters. Road distance is displayed on a different scale than the other covariates

The resource selection functions revealed distinct patterns of selection across hunting modes (Fig. 5, Additional file 1: Table S4). All hunters selected strongly and consistently for roads, as being necessary for mobility across the site. All hunters selected for areas with better views, with this pattern generally stronger for sit-and-wait and stalking hunters than coursing hunters, and stronger for successful hunters than unsuccessful hunters across all three modes. Stalking hunters avoided areas with dense shrub, with stronger avoidance in successful hunters. Both stalking and sit-and-wait hunters showed selection for more rugged terrain, with significantly stronger selection for successful stalkers than for unsuccessful stalkers.

## Discussion

The fine-scale movement tracking of 483 hunters revealed a diversity of successful hunting modes that mirrored those of non-human predators. Although all hunters used the same technique (rifle hunting) to harvest the same prey species (black-tailed deer) at a relatively small site in northern California, we identified distinct clusters of “sit-and-wait”, “stalking”, and “coursing” hunters. Differences in habitat selection across hunting modes indicate that there are distinct habitat domains associated with each mode, and differences in selection between successful and unsuccessful hunters suggest that hunting mode interacts with the physical landscape and other factors to shape patterns of harvest. Given this diversity of successful modes and habitat domains, contemporary hunters may create complex landscapes of risk for game species, making it difficult for prey species to effectively predict and respond to hunter behavior [25, 40].

Although hunters clustered into three distinct hunting modes (sit and wait, stalking, coursing), nearly all hunters exhibited each of the different behaviors (driving, walking, and remaining stationary) over the course of their hunt. Features of the physical environment predicted fine-scale movement decisions, as hunters transitioned between behavioral states as a function of landscape covariates. In areas with better views or more rugged terrain, for example, hunters were more likely to stop driving, and begin to walk or remain stationary. As the day progressed, hunters were more likely to drive relative to walking, potentially due to increased heat and exhaustion [41]. Technology, specifically access to guns and to vehicles, enables hunters to readily switch behaviors and modes, and the ease and regularity with which hunters move between behavioral states further adds to the complex landscape of risk for game species [25].

Importantly, we found differences in patterns of habitat selection among hunters of different modes, suggesting that hunting mode interacts with the physical environment to shape distinct habitat domains for coursing, stalking, and sit-and-wait hunters, a pattern well documented in non-human predators [16, 42]. Given that different landscape features facilitate different hunting strategies, it is unsurprising that patterns of habitat selection varied by hunting mode. The coursing hunters selected strongly for roads, which was unsurprising given that driving was their primary behavioral state. The stalking hunters avoided the dense chaparral, and although this very dense shrub provides suitable cover, it is nearly impossible to move through this habitat on foot, which explains why it was avoided by those who were walking.

Differences in habitat selection between successful and unsuccessful hunters hinted at ways in which the success of certain hunting modes may depend on the physical landscape [26, 31]. While all hunters selected for areas with better views, successful sit-and-wait hunters selected more strongly for areas with better views than unsuccessful sit-and-wait hunters, and it is possible that this strategy improved detection of prey. Successful sit-and-wait hunters also selected more strongly for areas with dense shrub, which provides cover and reduces detection of hunters by deer.

Notably, all successful and unsuccessful hunters across hunting modes selected very strongly for roads, and previous work from the study site found that roads were the most important predictor of deer harvest locations [30]. Given that roads provide access for hunters here and at many sites worldwide [43–45], there is a tight link between road networks and the spatial distribution of the lethal and non-lethal effects of hunting across the planet [26, 29, 32]. This strong association of roads and hunting has consequences for site-specific and regional game management, as road networks may offer managers a simple, easily identifiable proxy for hunting pressure, and road access can be managed to optimize the spatial distribution of hunting.

In addition to identifying distinct habitat domains across hunting modes, we also found differences in the timing of harvest across these modes, likely related to the interaction of hunting mode and deer diel activity patterns. Harvest by sit-and-wait hunters tended to occur early in the day, which could be explained by the crepuscular activity of deer, given that this hunting mode relies on deer movement in order to initiate a hunting opportunity [41]. There were relatively fewer deer harvested by stalking hunters later in the day, which may be related to the decline in walking behavior as the day progressed.

It is difficult to infer a causal relationship between habitat selection and hunting success, as it could be the case that more skilled and experienced hunters were both more likely to harvest a deer and more likely to select for certain features. For example, we found that successful stalkers tended to select for more rugged terrain further from roads as compared to unsuccessful stalkers. While it is possible that hunting on foot is more successful in rugged areas (perhaps because it is easier to sneak up on deer) and more successful away from roads (perhaps because deer feel safer and are less vigilant in more remote areas), anecdotal observations suggest that hunters with more skill and experience on the property are more inclined to walk on foot in challenging terrain far from the roads. Hunting experience broadly and site familiarity more specifically may both allow hunters to find and exploit areas with better habitat conditions for particular hunting modes. Ultimately, a hunter’s familiarity with the landscape was a more important predictor of harvest success than what hunting mode they used, as we found that multi-day hunters (who had been given a day to scope the property prior to hunting) were more successful than hunters who spent only a single day on the property. Additional data on hunter characteristics, such as age or mobility level, could help further refine models of behavior and strengthen causal inference, but such information was not available in the present study.

Ecological theory suggests that different hunting modes and habitat domains are likely to have different non-consumptive effects on prey [17, 42, 46]. Active hunting modes and large habitat domains, which result in more widely distributed risk cues, tend to be associated with weaker non-consumptive effects than sit-and-wait modes with more localized risk [25, 42]. In this study, however, the active hunting mode (coursing) was actually associated with a relatively narrow habitat domain, given the reliance on roads, which may make spatial patterns of risk more predictable for deer and thus elicit stronger responses. However, other adjustments in prey behavior may mitigate any costs of spatial responses to risk: given the relatively short time frame of the hunting season and the restriction of hunting to daytime hours, deer may adjust activity levels and diel activity patterns temporarily to mitigate risk, as we previously documented at this site [30]. Furthermore, humans are not a lethal threat to deer for most of the year, and deer may therefore habituate to the presence of humans and to vehicles and exhibit weaker behavioral responses. Thus, overall non-consumptive effects of hunting are unlikely to have significant effects on the deer population, in comparison to the large consumptive effects of harvest.

Given the important role of hunting both for ecosystems and human societies, improving our understanding of contemporary hunter behavior and its consequences for game populations and predator–prey interactions is an important domain for future research. Such insights may inform decisions about how to manage where, when, and how much hunting is allowed. Quantitative approaches to studying hunter movement could also be improved by exploring how hunter motivation, identity, and experiences [29, 32] and spatial and social interactions among hunters drive patterns of behavior, movement, and harvest, following similar research in wildlife ecology [47, 48]. Additionally, the simultaneous study of hunters and game species can shed light on predator–prey shell games, and the real-time spatiotemporal interactions that lead up to successful and unsuccessful hunts [33, 49, 50]. In our study, we were not able to evaluate how differences in deer activity and abundance across habitats ultimately shaped patterns of harvest, although deer behavior plays an equally important role as hunter behavior in determining hunter success.

## Conclusions

Advances in theory and methods in the study of predator–prey interactions have greatly expanded our understanding of predator hunting behavior in the last several decades, changing our understanding of consumptive and non-consumptive effects of predation [42]. By applying similar theories and techniques to human hunters across systems and contexts, there is an opportunity to vastly improve our understanding of the globe’s most significant apex predator. Our study offers new avenues for studying hunter behavior to better understand hunter space use and success. More broadly, this work exemplifies how the application of analytical approaches from animal movement ecology can shed light on the role of human behavior in socio-ecological processes. Similar approaches have been used to study movement syndromes of fishing vessels [51, 52]. Intraspecific variation in animal behavior and individual-level niche specialization is important to the stability of populations and ecological processes [53, 54], and our findings suggest that it may be similarly important to consider individual variation in human behavior in the context of ecological interactions. This mechanistic understanding of human mobility in the context of natural resource use can help us better predict and mitigate anthropogenic pressures on the environment.

### Supplementary Information

**Additional file 1**. Supplementary file containing detailed methods about data collection, processing, and analysis, and additional figures and tables that summarize the data and detailed results.

## Data Availability

A copy of data and code can be found on Dryad: https://doi.org/10.5061/dryad.000000083.
