# Supplementary material for: Hunting mode and habitat selection mediate the success of human hunters
Source: Mov Ecol. 2024 Apr 16;12:29. doi: 10.1186/s40462-024-00471-z (PMC11021010; doi:10.1186/s40462-024-00471-z)
Supplement: Supplementary file 1 — Additional file 1. Supplementary file containing detailed methods about data collection, processing, and analysis, and additional figures and tables that summarize the data and detailed results. [file 40462_2024_471_MOESM1_ESM.docx]

**Hunting mode and habitat selection mediate the success of deer hunters**

Kaitlyn M. Gaynor^*1^, Alex McInturff^*2^, Briana L. Abrahms^3^, Alison M. Smith^4^, Justin S. Brashares^5^

* authors contributed equally to this work; corresponding authors

**Affiliations**

^1^ Departments of Zoology and Botany, University of British Columbia, Vancouver, BC, Canada; kaitlyn.gaynor@ubc.ca

^2^ U.S. Geological Survey Washington Cooperative Fish and Wildlife Research Unit, School of Environmental and Forest Sciences, University of Washington; amcintur@uw.edu

^3^ Center for Ecosystem Sentinels, Department of Biology, University of Washington, Seattle, WA, USA

^4^ Hopland Research and Extension Center, University of California, Division of Agriculture and Natural Resources, Hopland, CA, USA

^5^ Department of Environmental Science, Policy, and Management, University of California - Berkeley, Berkeley, CA, USA

Supplementary Materials

Any use of trade, firm, or product names is for descriptive purposes only and does not imply endorsement by the U.S. Government.

**Supplementary Methods**

***Dates of study***

Our study took place at Hopland Research and Extension Center (HREC) in Mendocino County, California over seven hunting seasons, spanning 2015-2022 (Table S1, Figure S1). In 2015, 2016, and 2017, a public, lottery-based hunt occurred on six days, during the first three weekends (Saturday and Sunday) of the California Zone A hunting season in August. There was no hunt in 2018 due to a wildfire. In 2019, the public hunt occurred on only the first two weekends of the hunting season, and a limited two-day hunt for only two hunters was offered on the last weekend. In 2020-2022, the public hunt occurred on the second and third weekends of the hunting season, with a limited hunt offered on the first and last weekends of the hunting season (these limited hunts were two-day hunts in 2020, and three-day hunts in 2021 and 2022).

***Table S1***. Dates on which hunters were tracked at the Hopland Research and Extension Center in Mendocino County, California. NA = not available

| **Year** | **Dates of single-day public hunt** | **Dates of multi-day hunt** |
| --- | --- | --- |
| 2015 | August 8, 9, 15, 16, 22, 23 | NA |
| 2016 | August 13, 14, 20, 21, 27, 28 | NA |
| 2017 | August 12, 13, 19, 20, 26, 27 | NA |
| 2019 | August 17, 18, 24^1^, 25 | September 21-22 |
| 2020 | August 15, 16, 22, 23 | August 8-9, September 19-20 |
| 2021 | August 21, 22, 28, 29 | August 14-16, September 24-26 |
| 2022 | August 20, 21, 27, 28 | August 13-15, September 23-25 |

^1^ *No loggers deployed on August 24 2019, due to staffing constraints*

***Data cleaning***

Logger failure was higher in later years, due to the aging of our equipment (Figure S2). For the multi-day hunts, we collected loggers at the end of each day, and given that data were collected under the condition of anonymity we were unable to link tracks to a given hunter. We therefore treated each hunter-day as an independent track, and despite issues of pseudoreplication (for n = 31 of 483 tracks; Figure S2), we found no differences in any of our results when we ran the analyses for only the single-day hunts, and therefore feel comfortable that the pseudoreplication is not introducing bias into the study.

We clipped all tracks to the area in which hunting was permitted, to ensure we were capturing active hunting behavior. We resampled all tracks to a fix rate of 3 minutes using the amt::track_resample() function (R Core team 2023) to match our scale of inquiry and accommodate GPS error. We then removed any point that was >1.2 km from the previous point, which corresponds to a movement speed of at least 15 mph given a 3-minute fix rate. This is the speed limit in the study area, and also corresponds to a speed threshold at which drivers can no longer be considered to be actively hunting (searching for deer). This cut-off removed clear outliers due to GPS error or locations where the hunters were leaving the property at higher speeds. This resulted in the removal of 2,430 of 96,575 points (2.6%), leaving 94,145 steps for analysis.

While we did not formally interview hunters, our discussions with them helped refine our characterization of their movement behavior. In discussions with hunters at the study site, hunters reported that they were economical with their time spent on the site, spending little to no time on activities other than active hunting. This is unsurprising, given that the site is a highly desirable hunting location, and hunters were either limited to a single day at the site (public lottery hunt) or paid a large amount of money for the multi-day hunt opportunities. As a result, we assumed that time spent in the huntable zone was active hunting time. GPS points from the hunting-prohibited zone were removed from the analysis.

***Methods for developing spatial covariates***

We calculated ruggedness, which considers variability in both slope and aspect (as determined by the 10-meter USGS National Elevation Dataset) within a neighborhood using the Vector Ruggedness Measure tool for ArcGIS, which was adapted from Hobson 1972. We calculated this metric over a series of neighborhood sizes, from 900m2 to 12,100m2. Preliminary modeling indicated that a neighborhood of 900m2 resulted in the best-fit models of hunter behavior, and we therefore used this fine-scale measurement of ruggedness in all subsequent modeling.

To quantify the visibility hunters would have from any given point at the study site, we created a fishnet of points spaced 10 meters apart in a square grid across the extent of the study site. We used the Viewshed 2 tool in ArcGIS to identify the total amount of visible space from each point based on a 6-foot tall observer. We restricted this analysis to a 400m buffer from each point, as this is the maximum distance at which a hunter is likely to locate a deer (Higley 2002). We combined the data from these individual points into a raster layer indicating, for each cell, the total visible area to a hunter on the ground.

We created a fine-scale habitat map of the study area by manually digitizing remotely-sensed satellite imagery. We ground-truthed this map in 2015 by navigating to 50 random points and cross-validating their on-the-ground classification with the remotely sensed classification (49 of the 50 classifications matched, for an accuracy of 98%). For our analyses, we condensed habitat into three categories: grassland (20% of study area), woodland (41%), and chaparral (38%). We then calculated the percent habitat cover for each of the three habitat types throughout the study area, using a 150m radius. Grassland density and woodland density were correlated, and we dropped grassland density as preliminary modeling suggested that woodland density was a better predictor of hunter behavior.

**Supplementary Figures & Tables**

***Figure S1****. Sample sizes of hunter tracks per year in the Hopland Research and Extension Center study area in California, USA. The number of hunters permitted on the property varied year-to-year, and there was no hunt in 2018 due to fire. All hunters were provided with GPS loggers (with the exception of a single day in 2019), but higher failure rates in 2021 and 2022 resulted in lower sample sizes in these years. “Good track” indicates whether or not we had a usable GPS track for a hunter.*


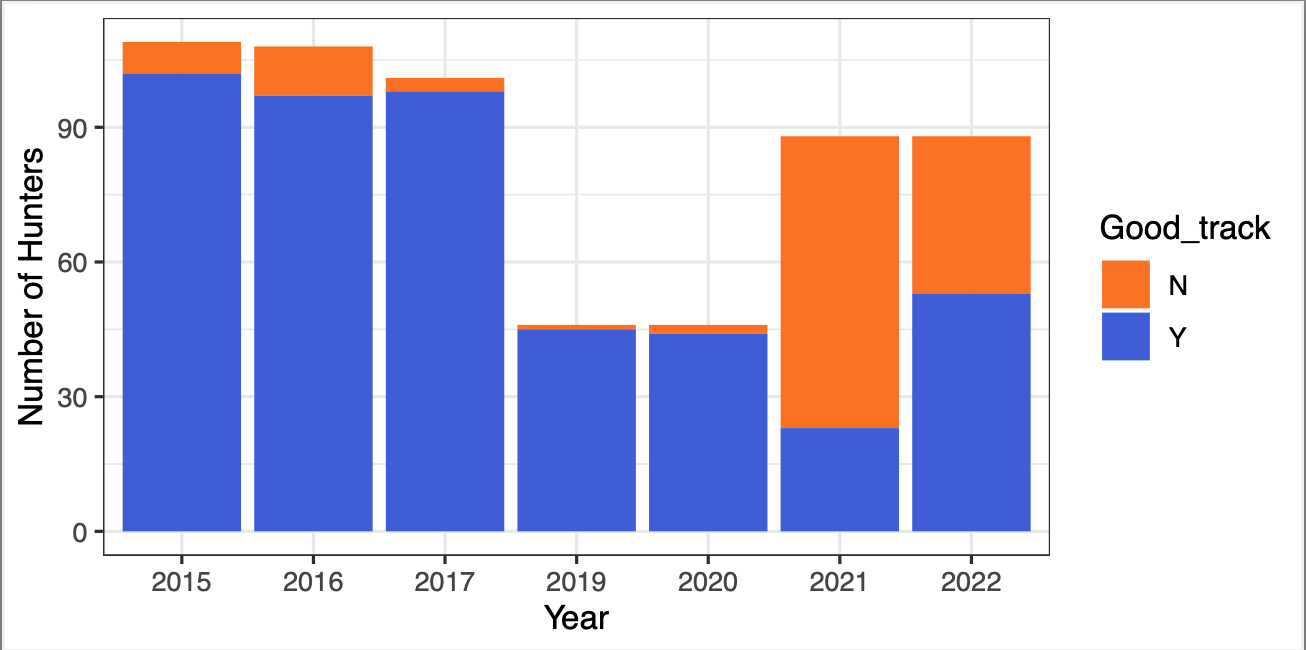


***Figure S2****. Sample sizes of usable hunter tracks from the Hopland Research and Extension Center in California, USA, for single-day and multi-day hunts. Hunters were typically only on the property for a single day. However, in 2019-2022, some hunters were permitted to return to the property for up to 3 days in a row (resulting in 31 non-independent tracks).*


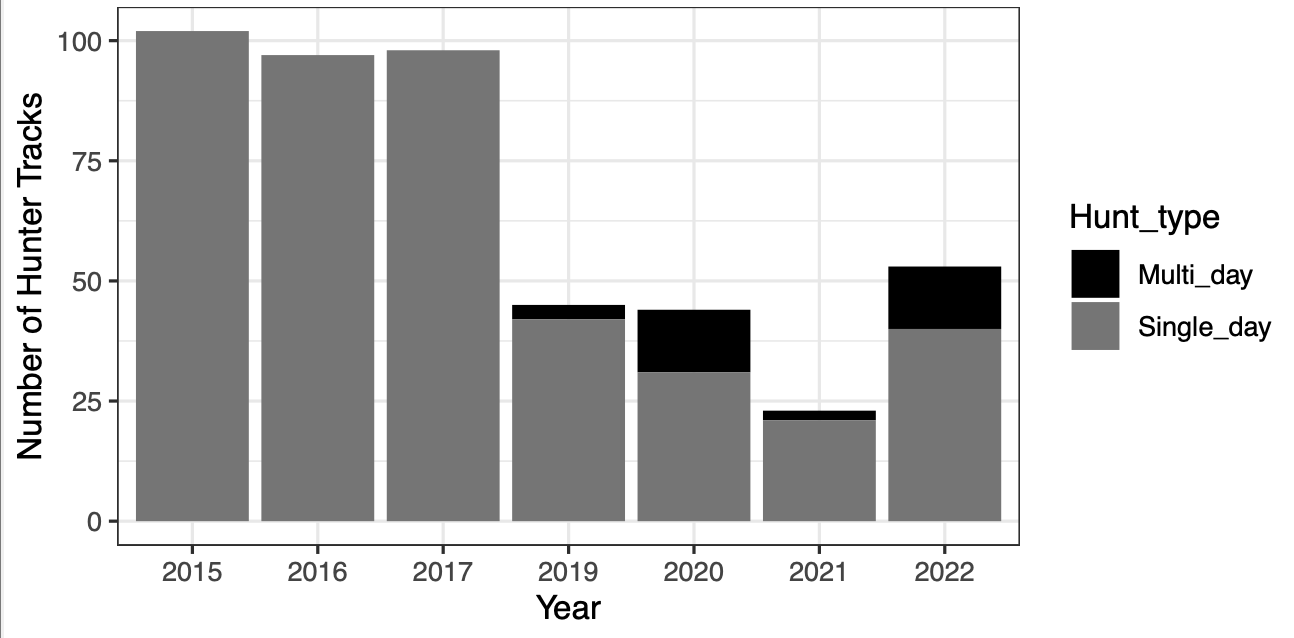


***Figure S3****. Distribution of fitted (A) step lengths and (B) turning angles for all hunter movement paths at the Hopland Research and Extension Center, California. Histograms represent underlying data distribution, and curves represent fitted densities for each behavioral state as determined by the Hidden Markov Model.*


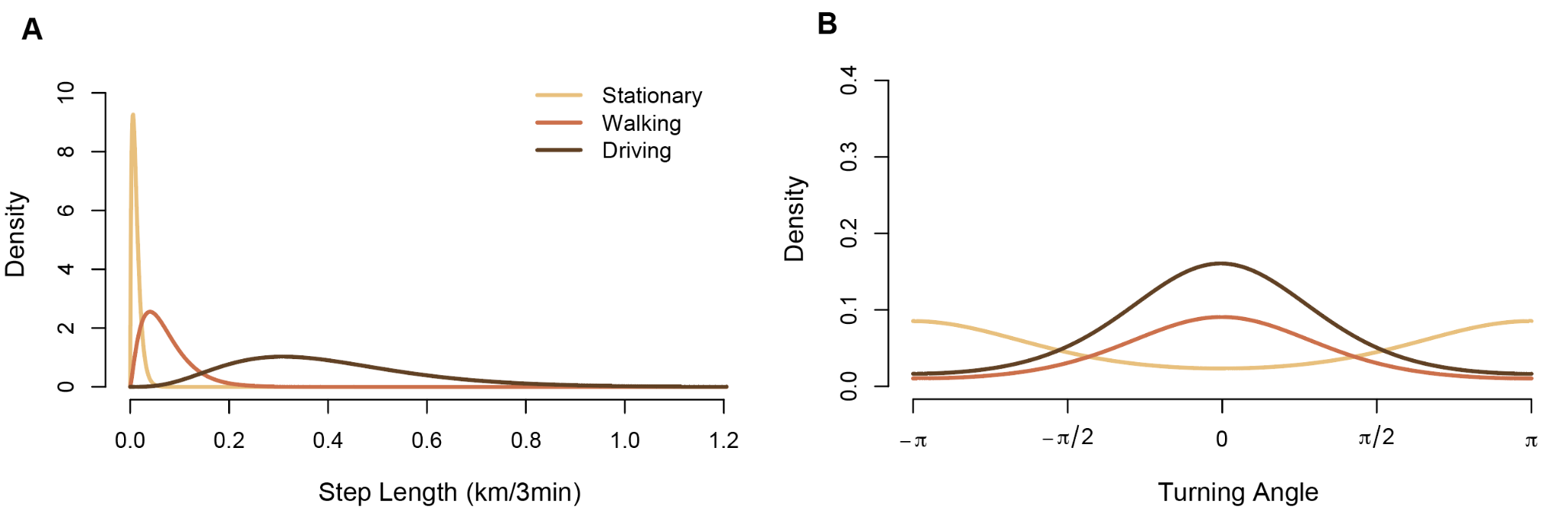


***Figure S4****. Histograms of distance to road for all hunter GPS locations that were classified as “stationary” by the Hidden Markov Model, displaying (A) all points and (B) only points within 50 meters of the road, for a finer-scale view. We classified all points within 10 meters of a road as “on road,” and all other points as “off road.”*


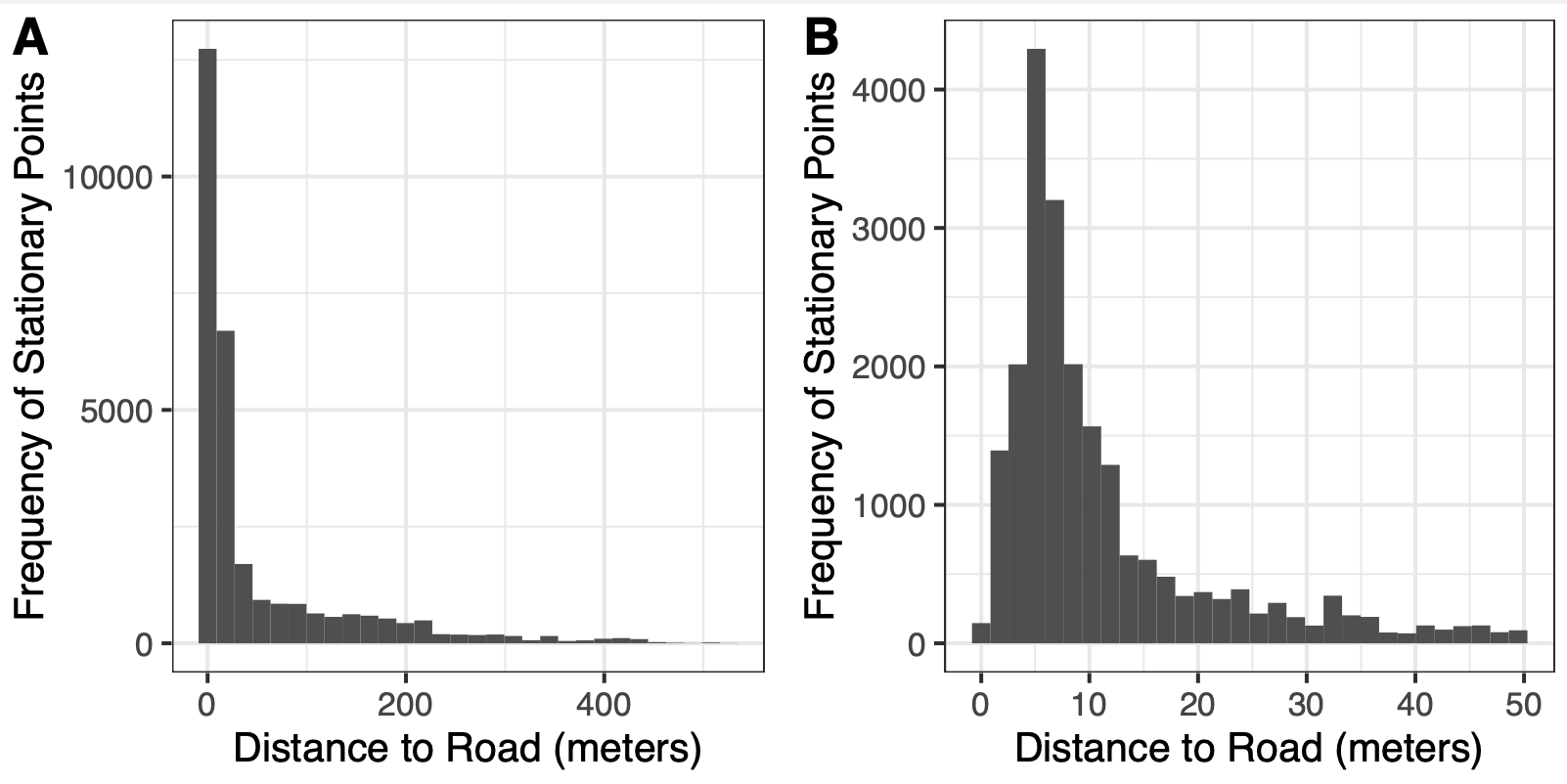


***Figure S5***. Beta coefficients and 95% confidence intervals for the effects of covariates on probability of transition between three hunter behavioral states (stationary, driving, and walking).

***
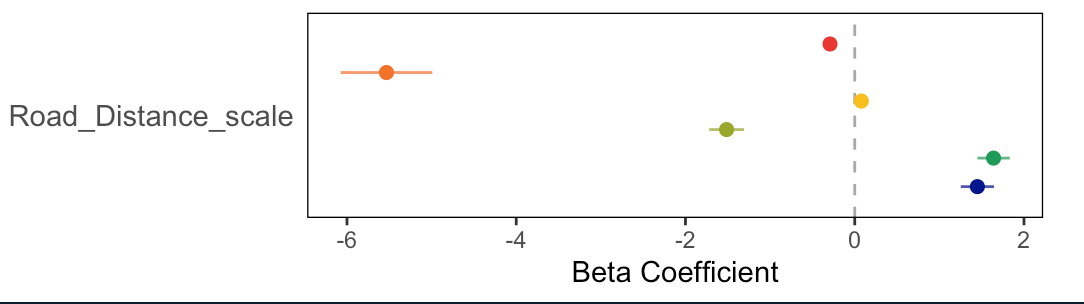

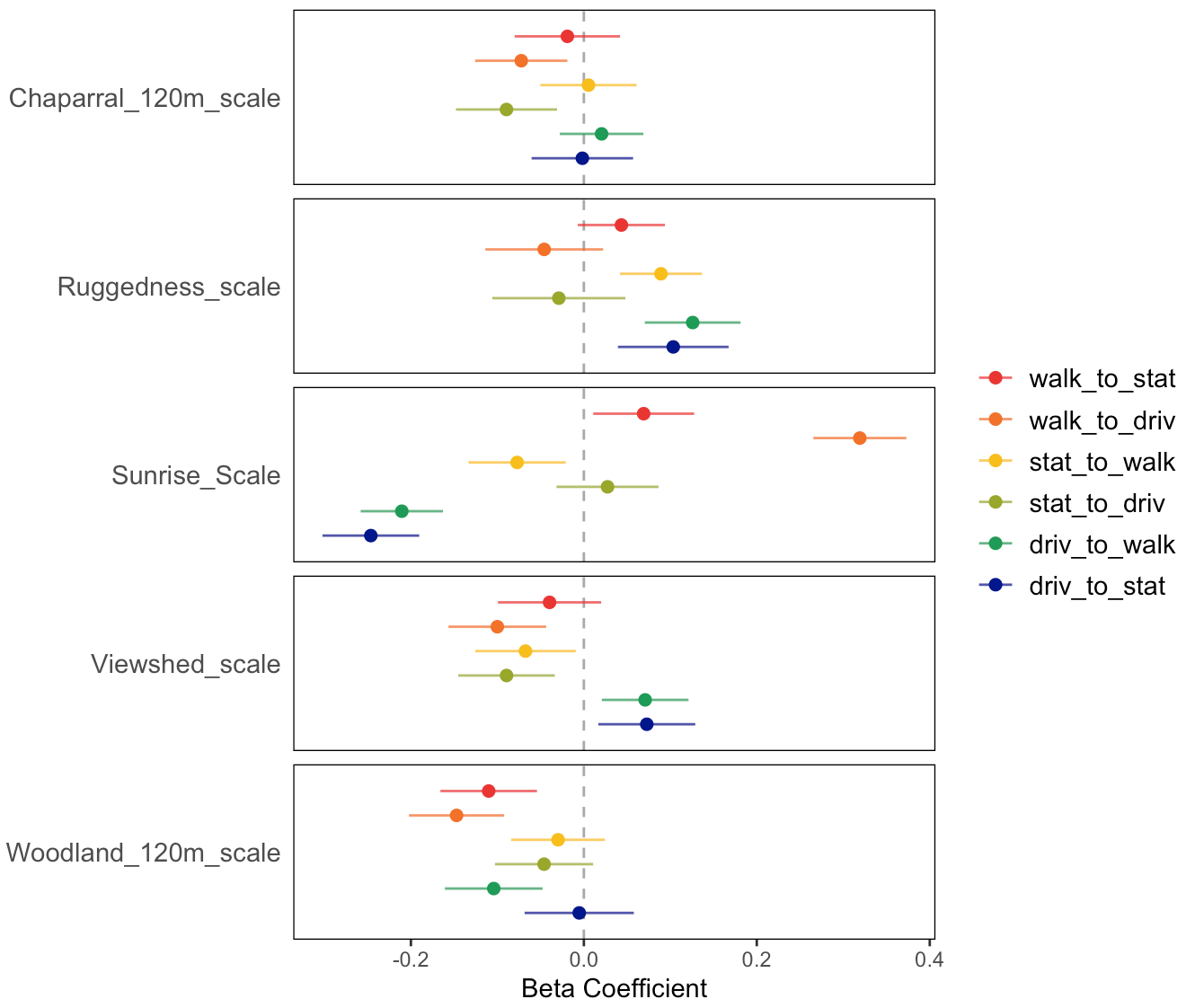
***

***Figure S6****. Stationary state probabilities as a function of covariates in the Hidden Markov Model. The lines (with 95% confidence intervals) represent the relative probability of a hunter being in a given behavioral state (stationary, walking, or driving) for covariate values.*


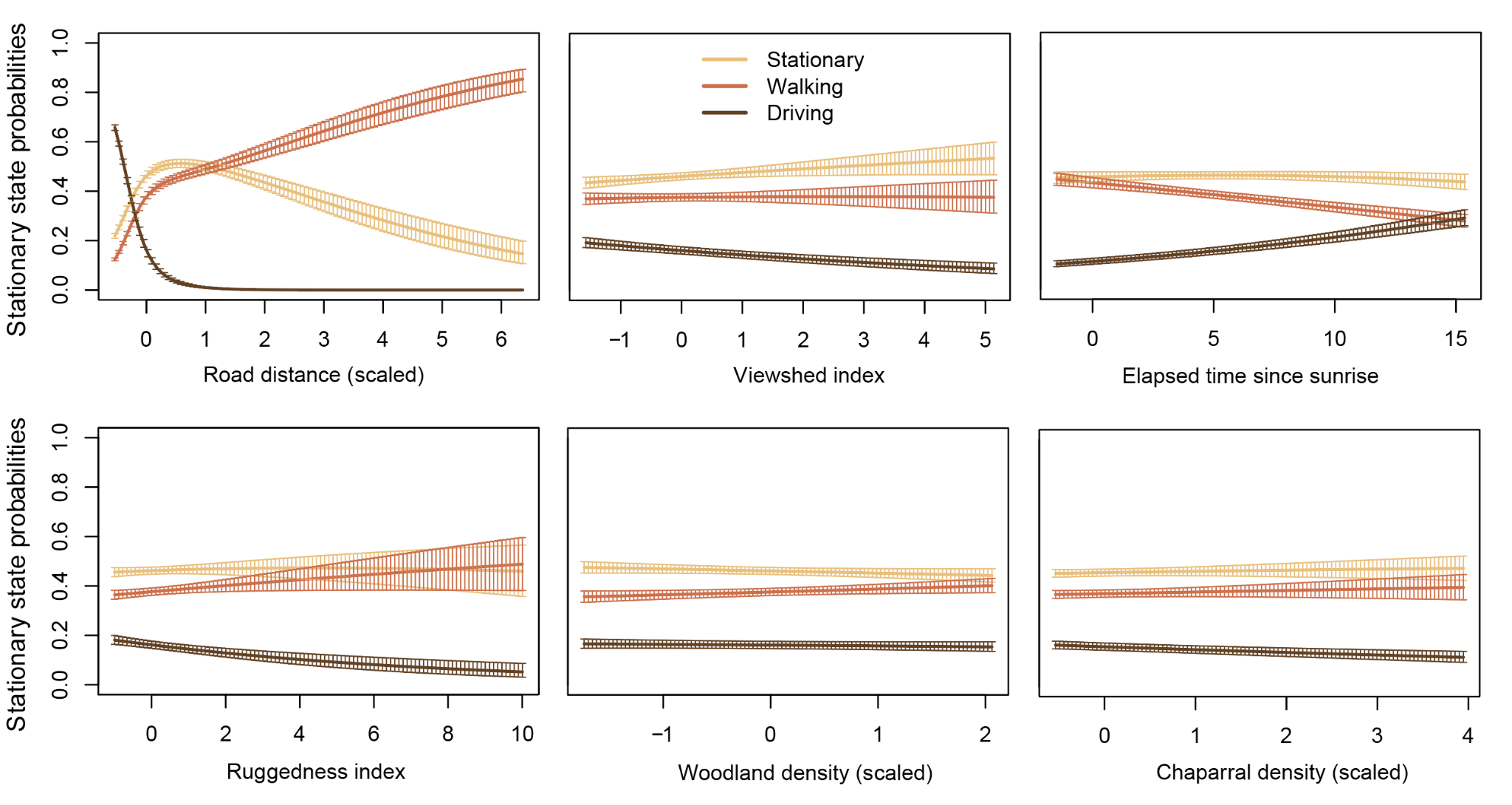


***Figure S7.*** *Sample tracks from hunters at the Hopland Research and Extension Center, California, USA including hunters that were classified as (A) sit-and-wait, (B), coursing, and (C) stalking. Points are colored by the most likely behavioral state as predicted by the Hidden Markov Model.*

**
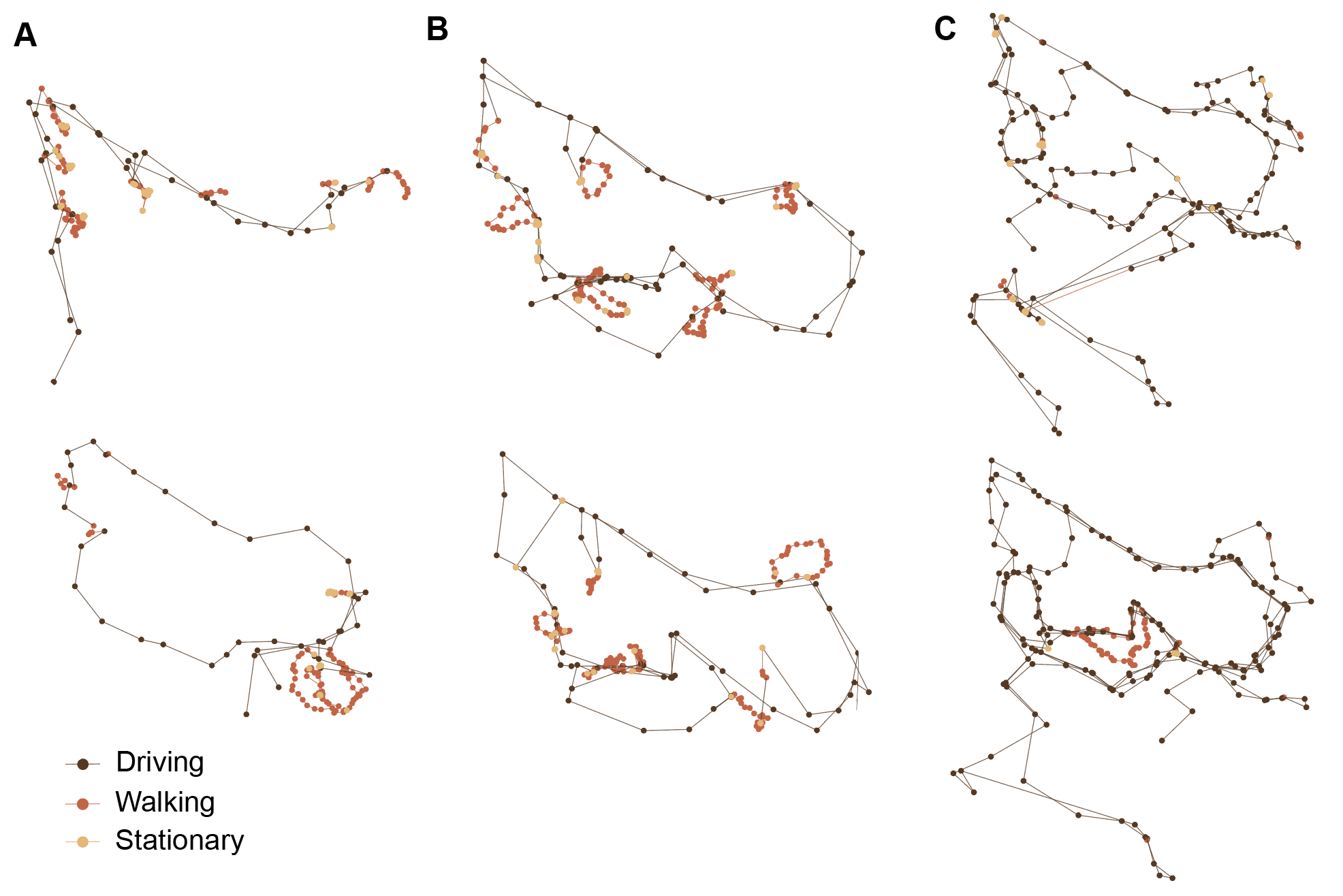
**

***Figure S8***. Breakdown of the relative time spent in each behavioral state, as identified by the Hidden Markov Models, for 75 randomly-selected hunters in each hunting mode.


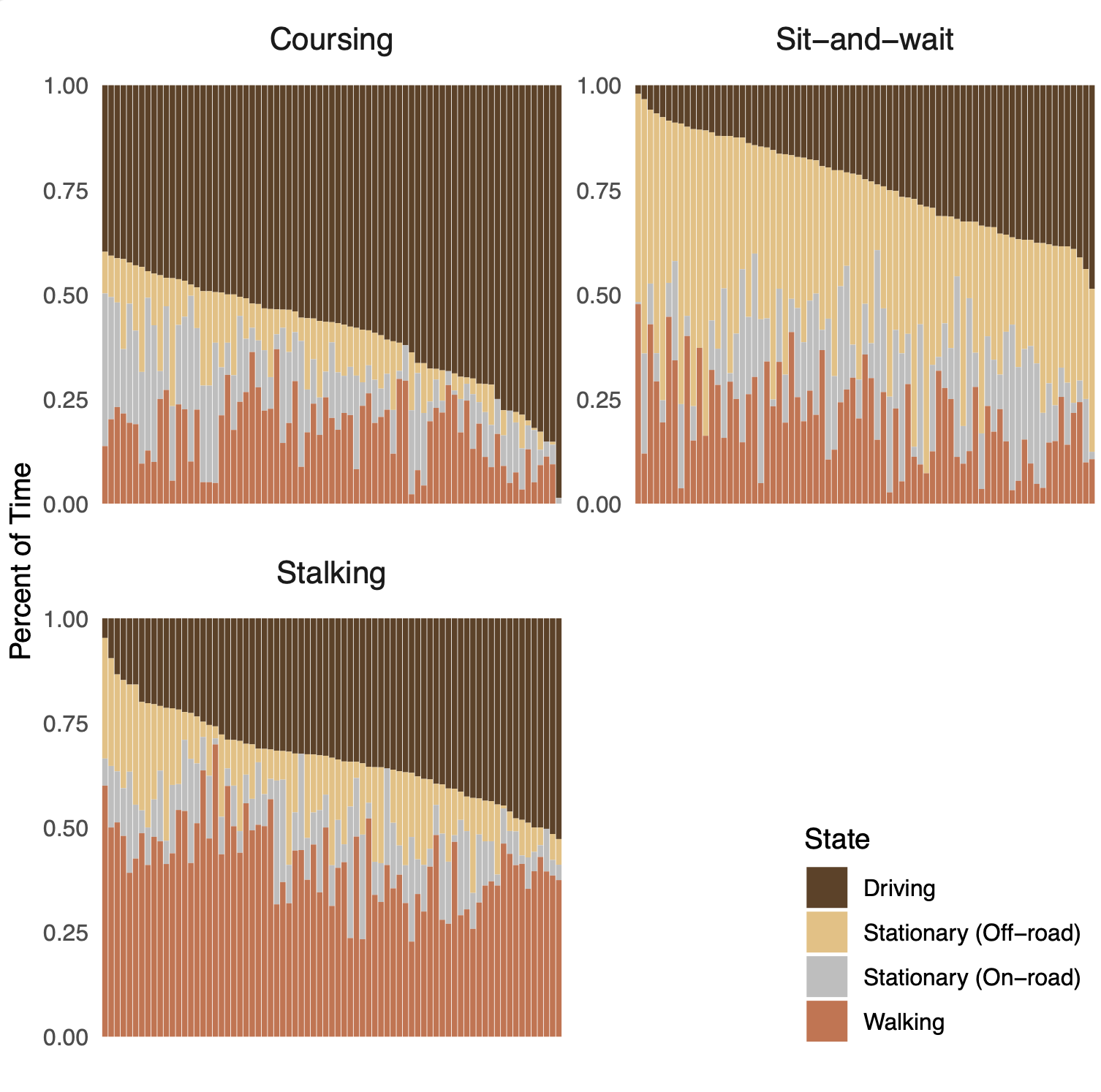


***Figure S9****. Factors predicting deer harvest success among hunters at the Hopland Research and Extension Center, California, USA. Plots show model predictions from the best model (lowest Akaike Information Criterion (AIC)) in which a variable appears. (A) The “coursing” hunting mode was marginally more successful than “sit-and-wait” or “stalking.” (B) Hunters that spent more than one day hunting on the property were more successful (on any given day) than hunters that only spent a single day hunting. (C) There was a slight trend towards increasing hunting success over time. Values in A and B are means and error bars are 95% confidence intervals. Shaded area in C is the 95% confidence interval.*


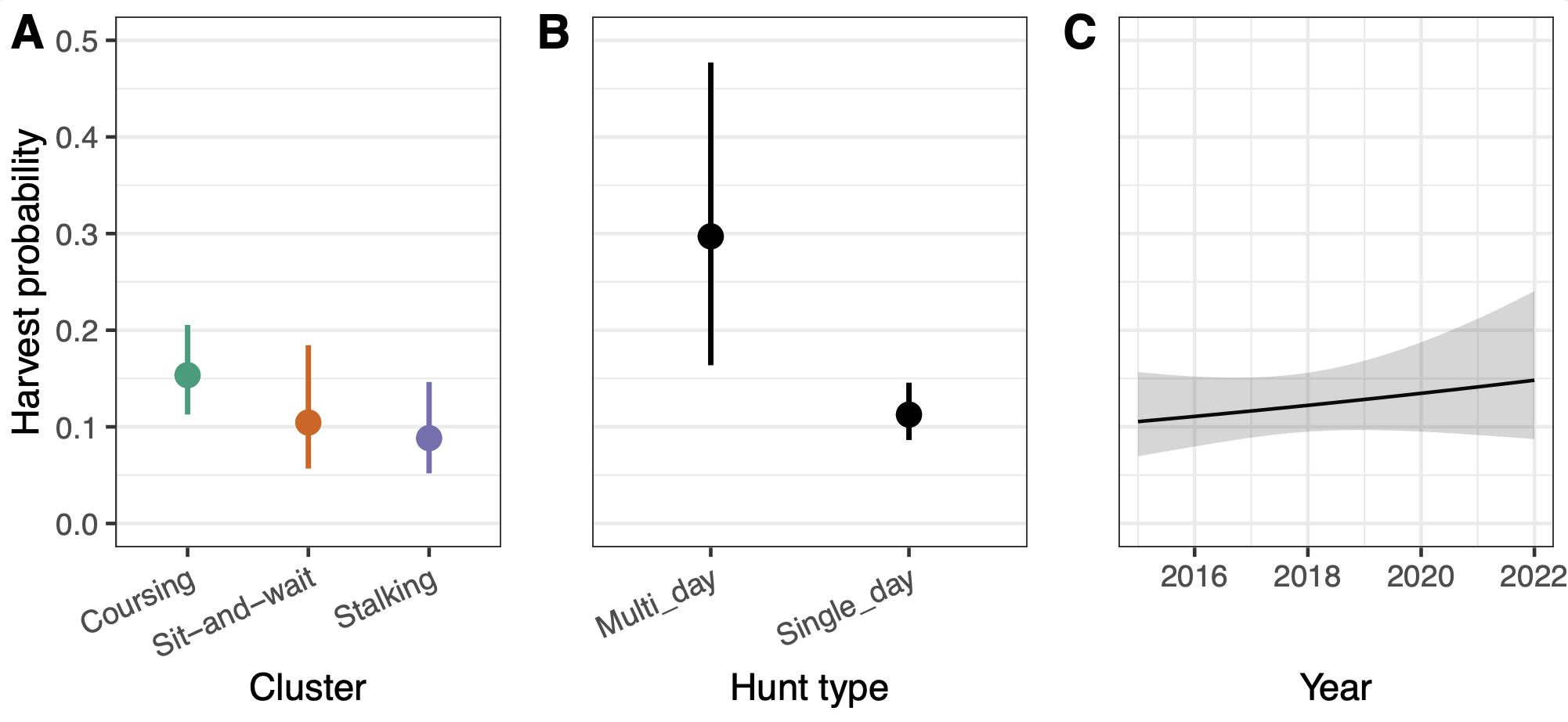


***Figure S10.*** *Habitat selection patterns of rifle hunters at the Hopland Research and Extension Center, California, using data at a 30-minute interval (rather than 3-minute interval, as presented in the main text). Points represent coefficients from Resource Selection Function (RSF) models (with 95% confidence intervals) for each of the three hunting modes (coursing, sit-and-wait, stalking), run separately for successful and unsuccessful hunters. Road distance is displayed on a different scale than the other covariates.*

**
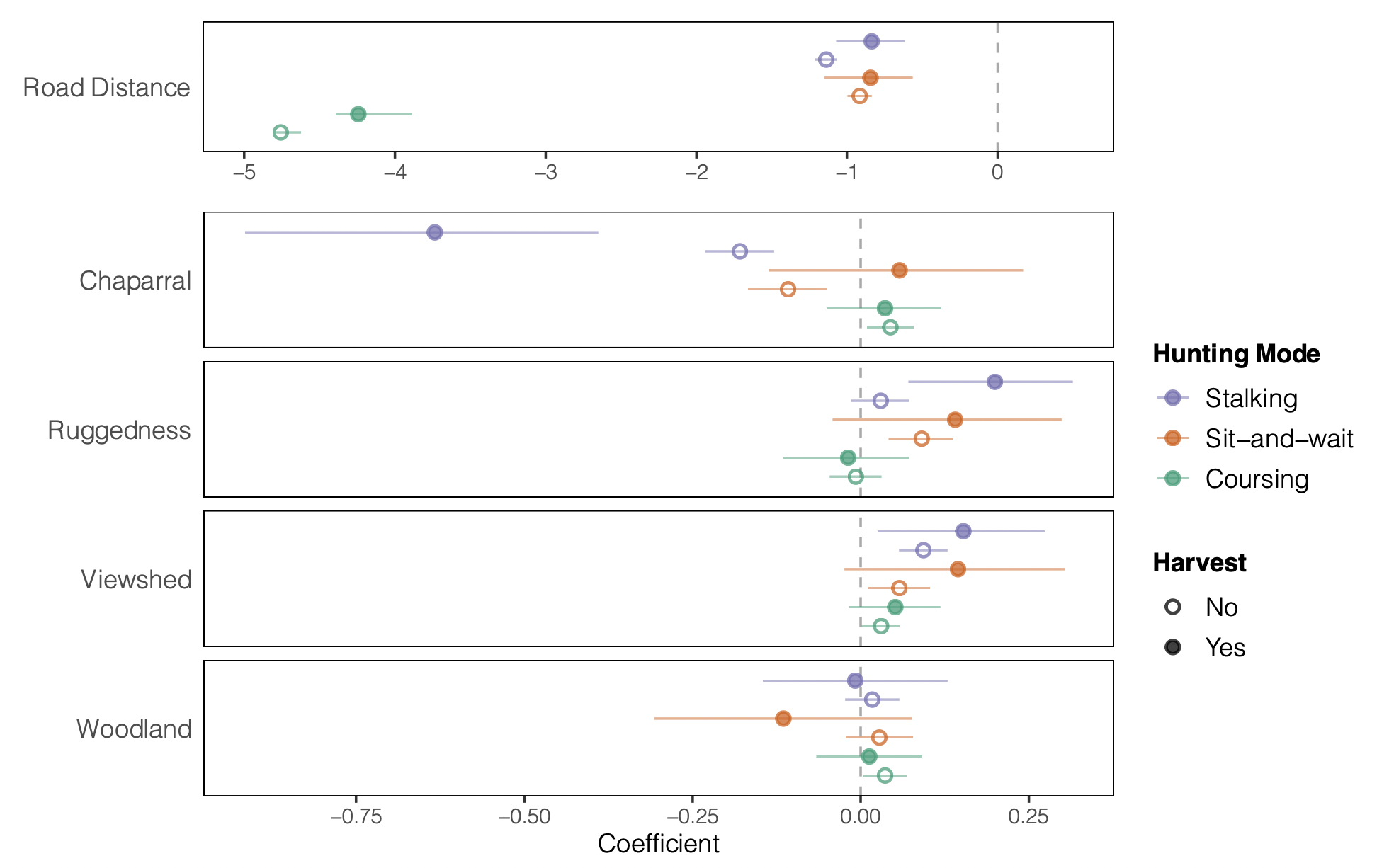
**

***Figure S11***. *Habitat selection patterns of rifle hunters at the Hopland Research and Extension Center, California, clustering hunters based on behavioral states predicted by an Hidden Markov Model with no environmental covariates (rather than from an HMM with environmental covariates, as presented in the main text). RSF model coefficients had a similar magnitude and direction as those reported in the model in the main text, with the exception of sit-and-wait hunters and chaparral (this changed here because different individual hunters were classified as sit-and-wait hunters). Points represent coefficients from Resource Selection Function (RSF) models (with 95% confidence intervals) for each of the three hunting modes (coursing, sit-and-wait, stalking), run separately for successful and unsuccessful hunters. Road distance is displayed on a different scale than the other covariates.*


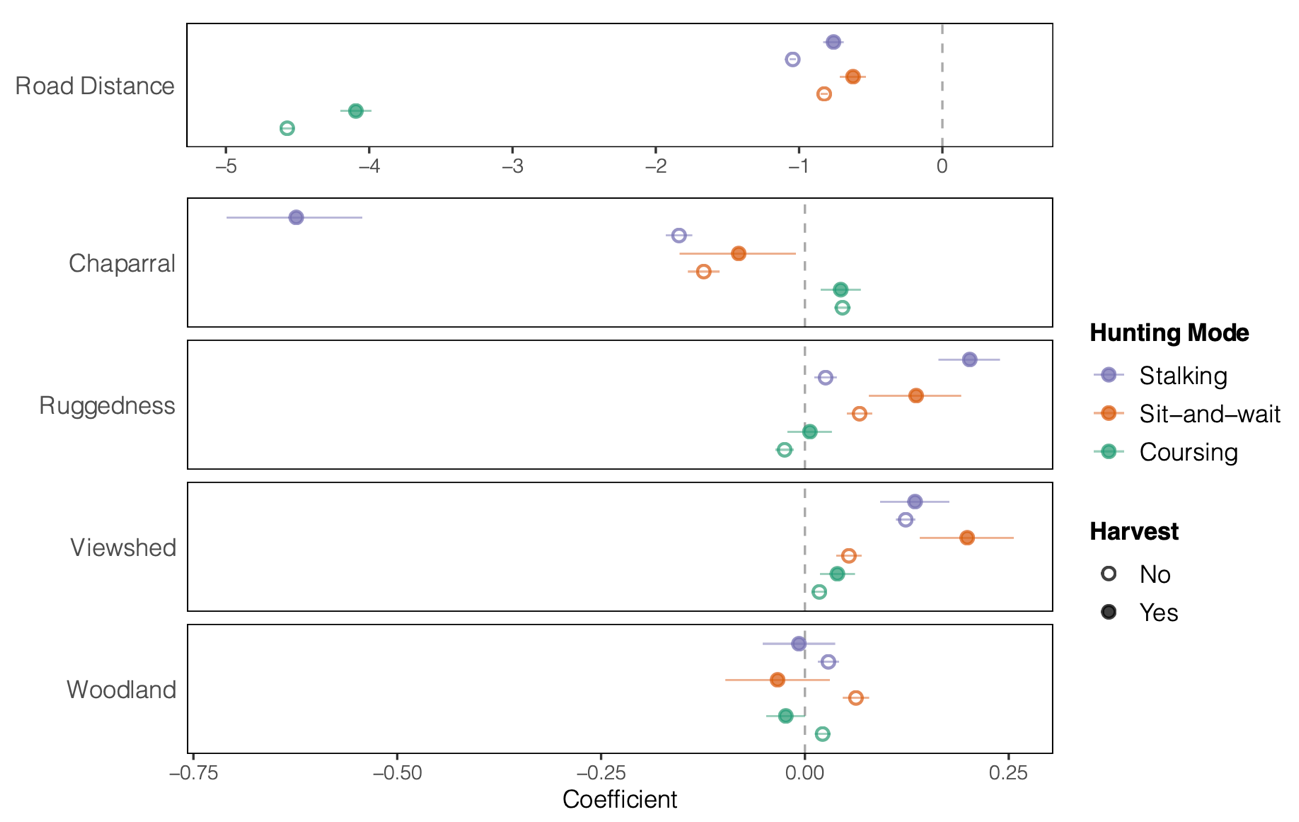


***Table S2****. Comparison of models predicting harvest success of hunters at the Hopland Research and Extension Center, California. Models are ranked from lowest to highest Akaike Information Criterion (AIC).*

| **Covariates** | **AIC** | $\Delta$**AIC** | **Akaike weight** |
| --- | --- | --- | --- |
| Hunting mode + Single/multi-day | 365.64 | 0 | 0.32 |
| Single/multi-day | 365.65 | 0.01 | 0.32 |
| Hunting mode + Year + Single/multi-day | 366.91 | 1.27 | 0.17 |
| Hunting mode * Single/multi-day | 369.05 | 3.41 | 0.06 |
| Hunting mode + Year | 369.56 | 3.92 | 0.05 |
| Year | 370.05 | 4.41 | 0.04 |
| Hunting mode | 370.98 | 5.34 | 0.02 |
| Null model | 372.23 | 6.59 | 0.01 |
| Hunting mode * Year | 372.99 | 7.35 | 0.01 |

***Table S3****. Results of Hidden Markov Model, which was used to identify distinct behavioral states of rifle hunters at the Hopland Research and Extension Center, California*. *Table presents the parameters estimated for step length and turning angle associated with each of the three states.*

| **State** | **Step length mean** | **Step length standard deviation** | **Step length zero-mass parameter** | **Turning angle mean** | **Turning angle concentration** |
| --- | --- | --- | --- | --- | --- |
| Stationary | 0.012 | 0.009 | 0.503 | 3.134 | 0.648 |
| Walking | 0.071 | 0.047 | 0.047 | -0.005 | 1.088 |
| Driving | 0.398 | 0.188 | 0.002 | -0.015 | 1.139 |

***Table S4.*** *Summary of coefficients for the resource selection functions for successful and unsuccessful hunters of different hunting modes at the Hopland Research and Extension Center, California using a fix interval of 3 minutes. The models were based on a ratio of 1 used : 10 available points.*

| ***Coursing hunters*** | | | | | | |
| --- | --- | --- | --- | --- | --- | --- |
|  | ***Successful*** | | | ***Unsuccessful*** | | |
| **Coefficient** | **Estimate** | **S.E.** | **P-value** | **Estimate** | **S.E.** | **P-value** |
| Intercept | -7.580 | 0.057 | <0.001 | -8.203 | 0.027 | <0.001 |
| Ruggedness | 0.014 | 0.015 | 0.35 | -0.025 | 0.007 | <0.001 |
| Viewshed | 0.036 | 0.011 | 0.001 | 0.023 | 0.005 | <0.001 |
| Chaparral density | 0.038 | 0.014 | 0.007 | 0.047 | 0.006 | <0.001 |
| Woodland density | -0.027 | 0.013 | 0.04 | 0.022 | 0.005 | <0.001 |
| Road distance | -4.066 | 0.057 | <0.001 | -4.692 | 0.027 | <0.001 |
| ***Stalking hunters*** | | | | | | |
|  | ***Successful*** | | | ***Unsuccessful*** | | |
| **Coefficient** | **Estimate** | **S.E.** | **P-value** | **Estimate** | **S.E.** | **P-value** |
| Intercept | -5.065 | 0.034 | <0.001 | -5.080 | 0.010 | <0.001 |
| Ruggedness | 0.208 | 0.020 | <0.001 | 0.024 | 0.007 | <0.001 |
| Viewshed | 0.124 | 0.021 | <0.001 | 0.104 | 0.006 | <0.001 |
| Chaparral density | -0.655 | 0.044 | <0.001 | -0.151 | 0.008 | <0.001 |
| Woodland density | 0.010 | 0.023 | 0.65 | 0.035 | 0.007 | <0.001 |
| Road distance | -0.829 | 0.037 | <0.001 | -1.115 | 0.012 | <0.001 |
| ***Sit-and-wait hunters*** | | | | | | |
|  | ***Successful*** | | | ***Unsuccessful*** | | |
| **Coefficient** | **Estimate** | **S.E.** | **P-value** | **Estimate** | **S.E.** | **P-value** |
| Intercept | -4.893 | 0.038 | <0.001 | -4.923 | 0.011 | <0.001 |
| Ruggedness | 0.097 | 0.030 | 0.001 | 0.069 | 0.008 | <0.001 |
| Viewshed | 0.195 | 0.026 | <0.001 | 0.059 | 0.008 | <0.001 |
| Chaparral density | 0.048 | 0.032 | 0.13 | -0.105 | 0.010 | <0.001 |
| Woodland density | -0.050 | 0.031 | 0.11 | 0.054 | 0.008 | <0.001 |
| Road distance | -0.807 | 0.048 | <0.001 | -0.907 | 0.013 | <0.001 |

***Table S5.*** *Summary of coefficients for the resource selection functions for successful and unsuccessful hunters of different hunting modes at the Hopland Research and Extension Center, California using a fix interval of 30 minutes. The models were based on a ratio of 1 used : 10 available points.*

| ***Coursing hunters*** | | | | | | |
| --- | --- | --- | --- | --- | --- | --- |
|  | ***Successful*** | | | ***Unsuccessful*** | | |
| **Coefficient** | **Estimate** | **S.E.** | **P-value** | **Estimate** | **S.E.** | **P-value** |
| Intercept | -7.672 | 0.183 | <0.001 | -8.138 | 0.084 | <0.001 |
| Ruggedness | -0.023 | 0.049 | 0.64 | -0.003 | 0.020 | 0.90 |
| Viewshed | 0.052 | 0.035 | 0.14 | 0.026 | 0.015 | 0.07 |
| Chaparral density | 0.033 | 0.044 | 0.46 | 0.040 | 0.018 | 0.03 |
| Woodland density | 0.020 | 0.041 | 0.63 | 0.033 | 0.017 | 0.05 |
| Road distance | -4.140 | 0.183 | <0.001 | -4.626 | 0.083 | <0.001 |
| ***Stalking hunters*** | | | | | | |
|  | ***Successful*** | | | ***Unsuccessful*** | | |
| **Coefficient** | **Estimate** | **S.E.** | **P-value** | **Estimate** | **S.E.** | **P-value** |
| Intercept | -5.058 | 0.106 | <0.001 | -5.087 | 0.031 | <0.001 |
| Ruggedness | 0.200 | 0.064 | 0.002 | 0.027 | 0.022 | 0.23 |
| Viewshed | 0.144 | 0.064 | 0.02 | 0.091 | 0.019 | <0.001 |
| Chaparral density | -0.634 | 0.134 | <0.001 | -0.183 | 0.027 | <0.001 |
| Woodland density | -0.015 | 0.071 | 0.83 | 0.018 | 0.021 | 0.39 |
| Road distance | -0.792 | 0.114 | <0.001 | -1.127 | 0.038 | <0.001 |
| ***Sit-and-wait hunters*** | | | | | | |
|  | ***Successful*** | | | ***Unsuccessful*** | | |
| **Coefficient** | **Estimate** | **S.E.** | **P-value** | **Estimate** | **S.E.** | **P-value** |
| Intercept | -4.896 | 0.119 | <0.001 | -4.921 | 0.033 | <0.001 |
| Ruggedness | 0.150 | 0.087 | 0.08 | 0.095 | 0.025 | 0.002 |
| Viewshed | 0.149 | 0.084 | 0.08 | 0.059 | 0.024 | 0.01 |
| Chaparral density | 0.071 | 0.097 | 0.46 | -0.109 | 0.031 | 0.004 |
| Woodland density | -0.102 | 0.099 | 0.30 | 0.025 | 0.026 | 0.33 |
| Road distance | -0.839 | 0.150 | <0.001 | -0.910 | 0.041 | <0.001 |
